# Qualitative and Quantitative Hippocampal MRI Assessments in Intractable Epilepsy

**DOI:** 10.1155/2013/480524

**Published:** 2013-07-29

**Authors:** Paramdeep Singh, Rupinderjeet Kaur, Kavita Saggar, Gagandeep Singh, Amarpreet Kaur

**Affiliations:** ^1^Department of Radiology, Guru Gobind Singh Medical College and Hospital, Baba Farid University of Health Sciences, Faridkot, Punjab 151203, India; ^2^Department of Medicine, Guru Gobind Singh Medical College and Hospital, Baba Farid University of Health Sciences, Faridkot, Punjab 151203, India; ^3^Department of Radiology, Dayanand Medical College and Hospital, Ludhiana, Punjab 141001, India; ^4^Department of Neurology, Dayanand Medical College and Hospital, Ludhiana, Punjab 141001, India; ^5^Department of Pediatrics, Guru Gobind Singh Medical College and Hospital, Baba Farid University of Health Sciences, Faridkot, Punjab 151203, India

## Abstract

*Aims.* To acquire normative data of hippocampal volumes and T2 relaxation times, to evaluate and compare qualitative and quantitative assessments in evaluating hippocampi in patients with different durations of intractable epilepsy, and to propose an imaging protocol based on performance of these techniques. *Methods.* MRI analysis was done in 50 nonepileptic controls and 30 patients with intractable epilepsy on 1.5T scanner. Visual assessment and hippocampal volumetry were done on oblique coronal IR/T2W and T1W MP-RAGE images, respectively. T2 relaxation times were measured using 16-echo Carr-Purcell-Meiboom-Gill sequence. Volumetric data was normalized for variation in head size between individuals. Patients were divided into temporal (*n* = 20) and extratemporal (*n* = 10) groups based on clinical and EEG localization. *Results.* In controls, right hippocampal volume was slightly more than the left with no effect of age or gender. In TLE patients, hippocampal volumetry provided maximum concordance with EEG. Visual assessment of unilateral pathology concurred well with measured quantitative values but poorly in cases with bilateral pathologies. There were no significant differences of mean values between extratemporal group and controls group. Quantitative techniques detected mild abnormalities, undetected on visual assessment. *Conclusions.* Quantitative techniques are more sensitive to diagnose bilateral and mild unilateral hippocampal abnormalities.

## 1. Introduction

Epilepsy is a familiar neurological disease characterized by recurrent seizures. Even though epilepsy is presently generally well manageable with modern antiepileptic drugs, there still remain about 30% of patients with epilepsy who do not respond to optimal treatment [1]. These patients are then understood to have intractable or medically refractory epilepsy. Most of the patients have had good outcomes after surgery, and this regularly depends on the presurgical evaluation by EEG and magnetic resonance imaging (MRI). Unilateral hippocampal sclerosis (HS) is the most frequent pathological finding in temporal lobe epilepsy (TLE), and up to 65% of cases of TLE can be attributed to pathology arising entirely in the hippocampus [2]. Visual (qualitative) assessment of T2-weighted changes (hyperintense signal on T2-weighted images and atrophy) was the earliest method that demonstrated an association among hippocampal pathology and MR-detectable signal abnormality. Hippocampal volume loss is a sensitive and specific pointer of hippocampal sclerosis in the clinical setting of epilepsy, and hippocampal volumetric study can quantify atrophy in TLE patients. T2 relaxometry is another quantitative technique to determine the frequency and severity of T2 abnormality. Hippocampal T2 relaxation time increases in patient of hippocampal sclerosis [3]. The aim of this study is to evaluate and compare the relative value of visual assessment, hippocampal volumetry and T2 relaxometry individually and in combinations, in the diagnosis of Hippocampal sclerosis. A crucial issue is whether these techniques provide complementary or superfluous information about the hippocampus. We also aim to provide normative MR hippocampal volumetric data in Indian population as well as to propose a multimodal MR imaging protocol based on performance of these techniques. 

## 2. Materials and Methods

This study included 30 patients with medically refractory epilepsy and 50 nonepileptic control subjects. Patients belonging to all ages and both sexes were included in this study. A detailed history was elicited from all patients. Positive and relevant past history was also recorded. All the patients were initially assessed clinically by a senior neurologist before referring them for MRI. All the MRI data was assessed by two senior radiologists. This project was reviewed and approved by the institutional review board, and all procedures were in agreement with institutional guidelines. 

## 3. Preparation of the Patient

Before starting the MR study, the procedure was explained to the patient in his/her vernacular language to allay the fear and anxiety. Length of the study in the magnet varied from 30 min to 45 min and was communicated to the patient before the start of study. During the entire period of procedure patient was in contact with technologist/doctor by a two-way intercom system. To reduce the artifacts due to patient movement, sedation was given to patients of the pediatric group and uncooperative patients.

## 4. MRI Assessment

### 4.1. Visual Analysis

MR imaging was performed on 1.5-T MRI scanner (Magnetom Avanto, 18 channels, Siemens Medical Solutions, Erlangen, Germany) with a matrix head coil used as both transmitter and receiver. T1W, T2W, diffusion-weighted, and HEMO sequences were obtained in axial plane with 5 mm slice thickness and 30% interslice gap. For dedicated hippocampal study, inversion recovery (IR) oblique coronal images (TE: 51, TR: 3500, FOV: 250 mm, slice thickness: 2 mm) and oblique coronal T2W images (TR: 4000, TE: 101, FOV: 230, slice thickness: 2 mm) covering the whole brain were acquired. Oblique coronal plane was perpendicular to the long axis of hippocampus. The images were assessed for hippocampal atrophy, loss of defined morphologic structure of hippocampus, increased T2W signal and decreased T1W signal. Apart from presence of severe atrophy, the diagnosis of the hippocampal sclerosis was made if there was also evidence of signal abnormality of the hippocampus. 

### 4.2. Volumetric Analysis

An oblique coronal three-dimensional gradient echo sequence (MP-RAGE: TR: 2400, TE: 3.75, FOV: 250, flip angle: 8, matrix: 192 × 192, slice thickness: 0.85 mm, interslice gap: 1.3 mm) was obtained perpendicular to long axis of hippocampus. Cross-sectional areas of both the hippocampi were measured in these oblique coronal sections by tracing hippocampal boundary manually from hippocampal head to tail. On hippocampal head area the CSF in the uncal recess of the temporal horn when visible was considered the most reliable boundary between the hippocampal head and the amygdala. If uncal recess was not visible, then the alveus was used. To standardize the measurement, first section of the anterior hippocampus was defined as point where the uncal recess or alveus first appears. Posterior margin of hippocampal volumetric measurement was defined by MR image where crus of fornix was seen in full profile. Lateral and medial borders were defined as CSF in temporal horn of lateral ventricle and CSF in uncal/ambident cisterns, respectively. Inferior border was defined by grey-white matter junction between subiculum and white matter of parahippocampal gyrus. The volumes of both hippocampi were calculated by summing each of the cross-sectional volumes {cross sectional area × (section thickness + interslice gap)}. Normal control values for hippocampal volumes were acquired from 50 control subjects using an identical protocol. Abnormal hippocampal volume values were considered when these were both outside the range of all normal control values and more than two standard deviations outside the mean value of control hippocampal volumes. Volumetric data was normalized for variation in head size between individuals by Gullap's formula [4]:

(1) $$\begin{array}{c} \text{NV}=\frac{\left(\text{MMAG}\right)\times \left(\text{HV}\right)}{\left(\text{MAS}\right)}, \end{array}$$

where NV is the normalized volume, MMAG the mean mid-sagittal area of the group (controls or cases), HV the absolute hippocampus volume, and MAS the mid-sagittal area of the subject.

### 4.3. T2 Relaxation Time Measurement

T2 relaxation times were calculated using 16-echo Carr-Purcell-Meiboom-Gill sequence which is a multiple spin-echo sequence (TE: 22–352, TR: 3000, slice thickness: 5 mm, FOV: 230). 16 separate spin-echo images were acquired for each oblique coronal slice at echo times ranging from 22 ms to 352 ms. The T2 maps were acquired using a computer program that fitted a single exponential to the signal intensity data from corresponding pixels from all 16 echoes. The T2 relaxation time was then computed for each pixel, and an image was constructed in which pixel intensity corresponded to the calculated T2 relaxation time. The mean hippocampal T2 relaxation time was calculated by manually marking a region of interest (ROI) in the largest possible circular area within the anterior, middle, and posterior sections corresponding to the three sections of the hippocampus designated as hippocampal head, hippocampal body, and hippocampal tail, respectively, while evading boundaries where partial volume effects with CSF might arise. 

Normal control values for T2 relaxation time were acquired from control subjects using an identical protocol. Abnormal T2 values were considered when these were both outside the range of all normal control values and more than two standard deviations outside the mean value of control hippocampal T2 relaxation times. 

### 4.4. Video-EEG Monitoring

24-hour video-EEG monitoring was carried out for every patient either before or after the MRI by the standard protocol. The electrodes were glued to the scalp of the patients. A camera was used to visually record the patient activity continuously while at the same time the EEG was recording the brain activity. We used a TV monitor with a split screen in the room of the patient; the screen showed EEG on one side and the video recording of the patient on the other side. EEG was analysed for temporal or extratemporal localization of epilepsy.

### 4.5. Statistical Analysis

The MRI findings were compared with clinical data and 24-hour video-EEG findings, and a diagnosis was made. Patients were divided into temporal (*n* = 20) and extratemporal (*n* = 10) groups based on clinical data and EEG localization. Comparison of values obtained for the patient and control groups was made. Results were evaluated by SSPS version 16.0 for windows (SSPS Inc., Chicago, IL, USA). Pearson's correlation coefficient and *t*-test were used wherever indicated. *P* value < 0.05 was considered to be significant. 

## 5. Results

The patient group included 30 patients (19 males, 11 females) with a mean age of 24.3 ± 13.8 years (range: 8–54 years). The control group included 50 subjects (27 males, 23 females) with mean age of 29.5 ± 16.3 years (range: 6–77 years). Out of 30 cases, 5 had risk factors for intractable epilepsy. Two patients (6.67%) had history of febrile seizures, two patients (6.67%) had history of head trauma, and one patient (3.33%) had history of herpes simplex encephalitis. 

### 5.1. Evaluation Using Visual (Qualitative) Analysis

Using visual assessment, unilateral increased signal intensity on T2W images with unilateral atrophy was found in fifteen patients out of a total of 30 patients. Out of the fifteen patients, right- and left-sided MR signs of HS were found in ten patients and five patients, respectively. Left-sided hippocampal atrophy without increased signal intensity was diagnosed in one patient. None of the patients had right hippocampal atrophy without T2 hyperintensity. MR signs of bilateral hippocampal atrophy/sclerosis were found in none of the patients. Increased hippocampal signal on T2W images without hippocampal atrophy was found in one patient (Tables 1(a) and 1(b)). 

In 13 cases, the hippocampus was judged to be normal on visual inspection of the MRI. Out of these thirteen cases, four cases had diagnosis of temporal lobe epilepsy on EEG. The remaining nine patents had diagnosis of extratemporal/unlocalized epilepsy on EEG.

Three (10%) out of a total of 30 subjects had evidence of dual pathology on visual inspection. One had hippocampal pathology associated with cortical dysplasia, another had associated grey matter heterotropias with hemiatrophy, and the third had encephalomalacia.

### 5.2. Quantitative Evaluation

#### 5.2.1. Normative Data in Controls

Volumes of right and left hippocampi were measured, and T2 relaxometry values were calculated from MRI images in 50 control subjects. Volumetric data was normalized for variation in head size between individuals by Gullap's formula. The mean mid-sagittal head circumference in 50 control subjects was 165 cm^2^ (136.5–199.4 cm^2^). There was positive correlation (*r* = 0.55, *P* < 0.05) between mid-sagittal head circumference and absolute hippocampal volumes; that is, absolute volumes of hippocampi increase proportionately with head size. Right and left hippocampal volumes in controls were found to be positively correlated (*r* = 0.94, *P* < 0.01). Right hippocampal volume was larger than left by a statistically nonsignificant amount (*P* = 0.31). The mean normalized volume of right and left hippocampi in controls was 3.73 ± 0.40 cc (range: 2.97–4.28 cc) and 3.60 ± 0.41 cc (range: 2.95–4.5 cc), respectively. The range of measured T2 relaxation values from controls was 101–125.6 ms with a mean of 112.6 ms and SD of 6.25. The mean hippocampal volume was slightly larger in males (3.88 cc) than females (3.69 cc) but no significant difference was found between them (*P* = 0.20). There was also no correlation of hippocampal volumes and T2 relaxometry values with age (*P* > 0.10). Hippocampal volume ratio was obtained by division of smaller to larger hippocampal volume. The ratio in 20 control subjects ranged from 0.91 to 1, and ratio of less than 0.91 was considered to be diagnostic of unilateral hippocampal atrophy. The mean for normalized right-left hippocampal volume difference in controls was 0.155 ± 0.105 cm^3^ (0–0.3 cc). All the values which fall outside the upper limit of the range (0.3 cc) were considered abnormal. 

### 5.3. Patients Groups

Based on all information including EEG monitoring and excluding all MRI details, 20 out of 30 patients had diagnosis of temporal lobe epilepsy (TLE). These 20 TLE patients were further divided into right TLE and left TLE groups based on side of EEG localization. EEG localized 4 cases of bitemporal origin. Out of these 4 cases, 2 had dominant focus on right side and were grouped under right TLE and 2 had dominant focus on left side and were grouped under left TLE. The third group was of 10 patients whose seizure foci were either outside the temporal lobe or had a multifocal origin or could not be localized. This group was named as extratemporal/unclassified (ET/UC) group. 

### 5.4. Group Comparisons

Comparisons of right and left hippocampal volumes and T2 relaxometry values of cases with control values were made. Group values ipsilateral and contralateral to seizure focus were analyzed and were compared to control group.

**Figure 1:**
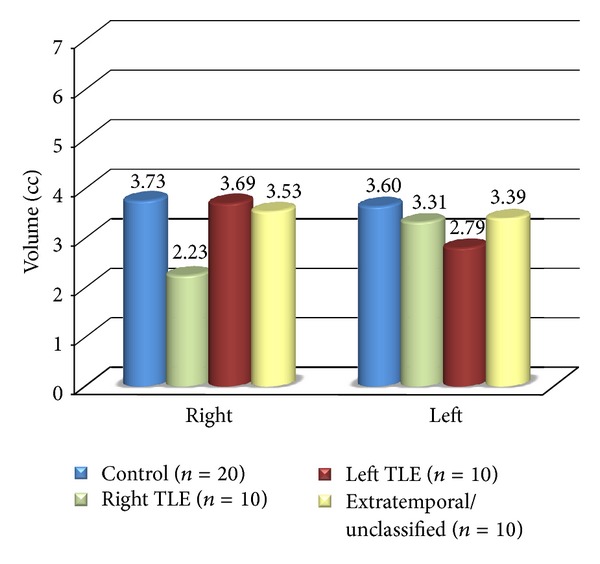
Mean hippocampal volumetry values in right and left hippocampi in controls and different groups.

**Figure 2:**
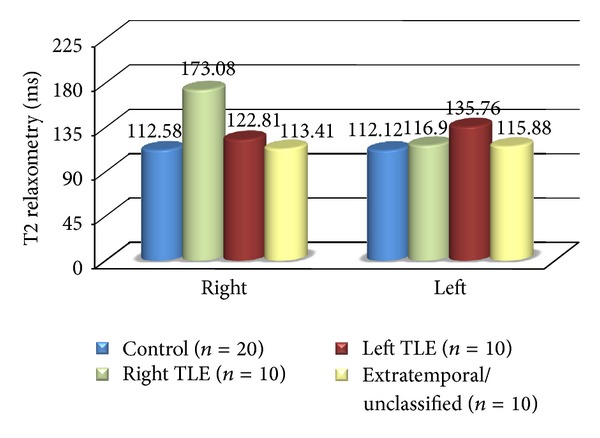
Mean T2 relaxometry values in right and left hippocampi in controls and different groups.

In groups with EEG focus lateralized to either left or right temporal lobe, both quantitative variables showed mean values that were significantly different to the controls in ipsilateral hippocampi (*P* < 0.01). Patients with intractable TLE had smaller mean hippocampal volume and longer T2 relaxation time ipsilateral to seizure focus (Table 2). Hippocampal damage in patients of extratemporal/unclassified group differed from that of patients with TLE. There was no significant difference (*P* > 0.10) in hippocampal volumes and T2 relaxation times between this group and controls (Figures 1 and 2). Hippocampal volumes and T2 values correlated inversely (*r* = −0.57, *P* < 0.01) with each other in TLE cases but not in extratemporal/unclassified group. 

## 6. Comparison and Correlation of Qualitative and Quantitative MRI Assessments in Cases of TLE (*n* = 20)

Visual assessment of unilateral volume loss (*n* = 13) concurred well with measured unilateral volume loss (*n* = 13) and with increased T2 values (*n* = 13), but visual assessment of bilateral atrophy (*n* = 0) concurred poorly with measured bilateral volume loss (*n* = 2) and with increased bilateral T2 values (*n* = 1) (Tables 3(a) and 3(b)). 

Of the 4 bitemporal cases diagnosed on EEG, 2 were diagnosed to have b/l hippocampal atrophy on volumetry but had normal T2 relaxometry values and normal signal intensity on T2W images. One patient had increased T2 relaxometry values bilaterally, but measured hippocampal volumes were normal, and in one patient hippocampal pathology was diagnosed only unilaterally by all three MRI modalities. 

18.75% (3 out of 16) of patients with unilateral TLE on EEG had either abnormal T2 values (*n* = 2) or abnormal hippocampal volume (*n* = 1) on the contralateral side in addition to abnormality present ipsilateral to seizure focus to EEG.

**Figure 3:**
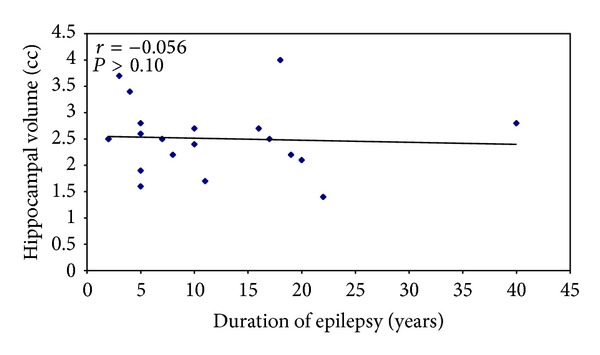
Relationship between duration of epilepsy and ipsilateral hippocampal volumes in TLE patients.

No significant correlation (*r* = −0.056, *P* > 0.10) was found between duration of epilepsy and hippocampal atrophy ipsilateral to seizure focus in both TLE and ET/UC groups (Figure 3), though there was mild reduction of ipsilateral hippocampal volumes with increasing duration of epilepsy in TLE group.

## 7. Comparison of Qualitative and Quantitative Assessments in Cases of ET/UC Group (*n* = 10)

No significant hippocampal abnormality was diagnosed on visual assessment in all the cases of ET/UC group. All cases were diagnosed to have normal hippocampal volumes and normal T2 signal intensity on visual impression. By contrast, hippocampal volumetry showed mild hippocampal atrophy in three patients (two bilaterally and one on left side). T2 relaxometry values were borderline in one patient. The rest of the patients had values within normal limits.

## 8. Hippocampal Volume Ratio (HVR) and Right-Left Hippocampal Volume Difference (HVD) in Controls and Cases

Hippocampal volume ratio (HVR) (obtained by division of smaller to larger hippocampal volume) and right-left hippocampal volume difference in controls and cases were calculated and were similar for both absolute as well as relative hippocampal volumes. HVR of less than 0.91 was considered to be diagnostic of unilateral hippocampal atrophy. HVD values which fell outside the upper limit of the range (0.3 cc) were considered abnormal. Using this criteria, 17 out of 20 (85%) TLE patients had volume difference of more than 0.3 cc and HVR of less than 0.91. Out of the 3 remaining patients, 2 had bitemporal abnormalities localized by EEG and had abnormalities in hippocampal volumetry or T2 relaxometry. The remaining patient had EEG localization in the left temporal lobe. Both qualitative and quantitative MRI techniques were not able to localize it (Tables 4(a) and 4(b)). 

One patient (10%) in ET/UC group had volume difference of more than 0.3 cc and HVR of less than 0.91. EEG was not able to localize the lesion in this patient, but hippocampal volumetry showed evidence of left hippocampal atrophy. T2 relaxation values were normal. The rest of the nine patients (90%) had HVR more than 0.91. 

## 9. Discussion

One of the objectives of the study was to give normative data that would be applicable to management of Indian patients with medically refractory epilepsy. The rationale accounts for the age range of 20–40 years in which maximum controls (22) of our study were there. This is the usual range in which surgery of these patients is considered. To our knowledge normalized MR hippocampal volumes in Indian population except pediatric population have not been reported so far. This normative volumetric data can be used in neuropsychiatric research and to evaluate hippocampal sclerosis in Indian population.

There has been extensive discussion relating to the variety of methods of MR-based volume estimates of the hippocampi. Obviously with different imaging parameters and quantification techniques, different results would be expected. We used the technique of Van paesschen [5] who reported a mean volume of 3.32 cc for left hippocampus and 3.33 cc for right hippocampus in his study. The normative findings of current study are slightly larger than these, 3.73 cc for right and 3.60 cc for left, as we adopted posterior boundary of Watson et al. [6], as it also allowed us to measure tail of the hippocampus. All the intervening slices were measured. Also, racial differences may account for some variation.

Assessment of 50 control subjects reconfirmed a few main trends reported earlier [7–13]. There was no significant association of age and sex with the size of hippocampus. There was positive correlation between right and left hippocampal volumes, and the right hippocampus was larger than the left by a statistically insignificant amount. Absolute Hippocampal volumes increased proportionately with total intracranial volume. Thus in order to directly compare values between different subjects, individual hippocampal volumes must be normalized. On the other hand, we also found that derived measures like hippocampal volume ratio (HVR) and R-L hippocampal volume difference are independent of total intracranial volume and remove the requirement for normalizing the data for intersubject variation. Though these measures helped us to distinguish between temporal and extratemporal epilepsies, but these were not able to recognize the bilateral hippocampal pathologies.

In the present study, we used multimodal set of MRI methods (visual analysis, hippocampal volumetry, and T2 relaxometry) to evaluate the occurrence and severity of hippocampal pathology in 30 patients (20 TLE and 10 extratemporal or unclassified). We further aimed to propose comprehensive MRI protocol for diagnosis of TLE patients. EEG and MRI data are autonomous sets of variables used to exemplify diverse abnormalities of the region of brain from where seizure initiates. Therefore, concordance of the results given by these techniques further authenticates the diagnostic conclusions derived from individual studies. On the other hand, one must be aware that one is likely to come across incongruent data between EEG and MRI, and in different conditions either one may be more problem-solving regarding identification of true epileptic focus. Preferably, one would want to evaluate the data given by MRI in patients in whom surgery had resulted in constant freedom from seizures and in those cases in which seizures were not cured after surgery. This was not practicable in present study because of long duration of follow-up period required.

Hippocampal volumetry provided 75% concordance with EEG and was the most sensitive of all techniques in detecting hippocampal pathology. Compared to controls, TLE patients had a smaller ipsilateral volume and an insignificant trend towards smaller contralateral volumes. Quigg et al. [14] stated that most patients (18%) with mesial temporal lobe epilepsy (MTLE) have some degree of bilateral, asymmetric hippocampal atrophy. In our study, 16.7% (5 of a total of 30) patients showed evidence of bilateral atrophy on volumetric analysis.

Overall, patients with extratemporal group did not show any considerable atrophy and may be judged to represent disease controls for this study. Adam et al. [15] stated that mild hippocampal atrophy (29%) was rarely associated with extratemporal epilepsy. In our study, three patients from ET/UC group (30%) had mild hippocampal atrophy which was detected by hippocampal volumetry and may represent such cases. 

Theodore et al. [16] found that epilepsy duration did have a significant effect on severity of hippocampal atrophy. However in our study, we also found no correlation between duration of seizures and hippocampal atrophy in both temporal and extratemporal epilepsy groups. One possible explanation for the difference in our results is that we did not take into account the frequency of seizures; more frequent seizures may have a positive correlation with duration of seizures. Also, because of small sample size of our study, definitive conclusions could not be made.

The range of measured T2 relaxation values from 50 healthy subjects was 101–125.6 ms with a mean of 112.6 ms and SD of 6.25. The method used for T2 relaxometry was similar to that described by Jackson et al. [17] with some modifications due to time constraints. It must be realized that these T2 values were empirically derived from data acquired under standard settings on our system and these values may vary on other systems. Unlike T1 values, T2 values are not considerably dependent on field strength. The capability to distinguish normal from abnormal will not vary but every centre must standardize the range of normal T2 values before deducing hippocampal pathology. 

Our T2 relaxation values in TLE corresponded to extent of severity of hippocampal damage which was agreed by volumetry to characterize hippocampal sclerosis. In our study, hippocampal T2 relaxation times correlated inversely with hippocampal volumes in TLE patients, but no correlation was found in extratemporal/unclassified group. It is likely that increasing hippocampal T2 relaxation time be a sign of more severe degree of damage. In patients with TLE, 70% had ipsilateral T2 relaxation time more than 126 ms in our study. Hippocampal T2 values were never greater than 126 ms in extratemporal/unclassified group. Therefore, T2 values >126 ms correlated strongly with the presence of hippocampal sclerosis in our study. In study done by Jackson et al. [17] 79% of the patients with TLE had abnormal ipsilateral hippocampal T2 values (T2 > 106 ms), and 65% of these patients had markedly elevated T2 values (T2 > 126 ms). This subset of patients had pathological evidence of hippocampal sclerosis.

Visual grading of unilateral hippocampal sclerosis was in agreement with volumetric and T2 measurements in lateralizing seizure disorder in 13 of 20 patients (65%). In our study, we found that all three modalities had equal efficacy in diagnosing unilateral pathology, but quantitative measures were superior in detecting bilateral and mild abnormalities. Since quantitative techniques are more sensitive to diagnose bilateral and mild unilateral hippocampal atrophy than qualitative techniques, these may reduce the need for invasive monitoring.

Lehéricy et al. [8] demonstrated that 8 out of 122 patients with hippocampal sclerosis had increased signal on T2W images without visually detectable atrophy. Similarly in our study, visual analysis revealed increased hippocampal signal on T2W images without atrophy in one patient. Another patient with bitemporal epilepsy on EEG had bilateral mildly increased T2 relaxometry values without measurable atrophy. However, it is difficult to label these cases as HS in absence of histopathological data. 

18.75% of TLE patients in our study had either increased contralateral T2 relaxometry values or abnormal contralateral hippocampal volume in addition to abnormal ipsilateral seizure focus on EEG. These may represent bilateral hippocampal abnormalities described in pathology studies by Margerison and Corsellis [2].

Proportion of patients with hippocampal sclerosis in our study may be underestimated as some of the patients may have hippocampal sclerosis without atrophy or increased signal on T2W images. Jackson et al. [18] reported 6 cases with lateralized intractable TLE in whom visual and quantitative MRI analysis revealed no hippocampal atrophy but in whom pathologically verified diagnosis was made. In our study, we had one patient in left TLE group in whom both qualitative and quantitative MRI analyses were normal. This may represent one such case. More detailed investigations such as invasive recording or PET may show the exact localization of seizure focus. Carne et al. [19] studied patients with TLE who had electroclinically well-lateralized temporal lobe seizures but had no evidence of hippocampal sclerosis (HS) on MRI. Many of these patients had concordant hypometabolism on fluorodeoxyglucose-PET ([18F] FDG PET). Based on the findings, they concluded that HS-negative, PET-positive TLE may be a surgically remediable syndrome distinct from HS positive TLE, with a pathophysiological basis that primarily involves lateral temporal neocortical rather than mesial temporal structures. Van Paesschen et al. [20] concluded that end-folium sclerosis or amygdala sclerosis should be considered in patients with TLE who have negative MRI. Alternatively, the lesion may be beyond the sensitivity of MRI. In our study, two patients in extratemporal/unclassified group had normal MRI with seizure focus (as localized by EEG) in frontal lobe. Therefore, there may be neuronal abnormalities which cannot be detected using MRI even with an optimum protocol.

**Figure 4:**
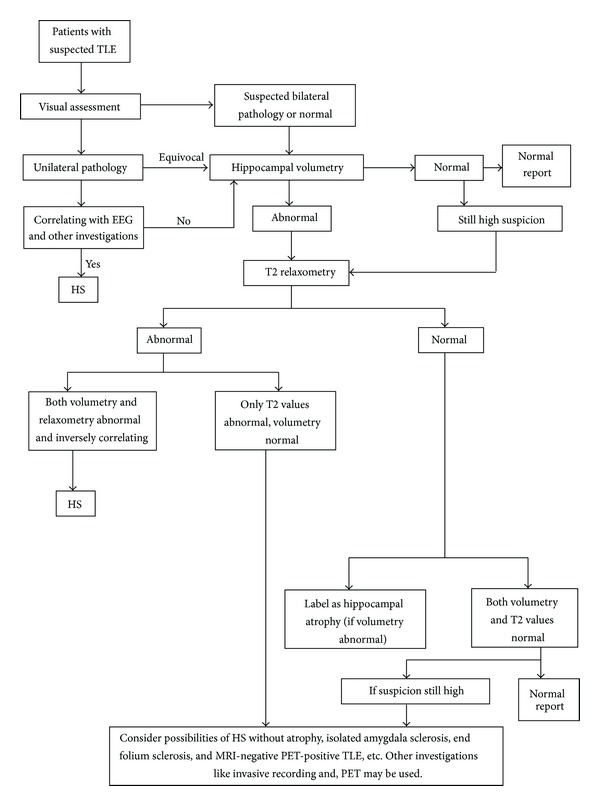
Proposed imaging protocol for intractable TLE patients.

One key objective of this study was to describe a simple multimodal MRI step-by-step protocol (Figure 4) to be implemented on daily routine. This protocol privileges visual assessment as the initial diagnostic method to study the hippocampal pathology, reserving hippocampal volumetry for diagnosing suspected bilateral and mild unilateral pathologies. Hippocampal volumetry when performed should be correlated with T2 relaxometry and EEG for the final diagnosis. Quantitative techniques should also be used in cases with negative qualitative assessment but with high suspicion of TLE and in cases with long history of intractable epilepsy. One should keep in mind the possibilities of HS without atrophy, isolated amygdala sclerosis, end-folium sclerosis, and MRI-negative PET-positive TLE in cases where MRI is negative and is not correlating with EEG findings. Other investigations such as nuclear imaging and invasive recording may be opted in such cases. 

## 10. Summary and Conclusions

In control subjects, absolute hippocampal volumes increase proportionately with head size. Right and left hippocampal volumes are positively correlated, and right hippocampal volume is larger than left by a statistically insignificant amount. No significant correlation of hippocampal volumes and T2 relaxation times exists with gender or age. 

Patients with intractable temporal lobe epilepsy have smaller mean hippocampal volume and longer T2 relaxation time ipsilateral to seizure focus. There is no considerable difference in hippocampal volumes and T2 relaxation times between extratemporal/unclassified group and controls. Hippocampal volumes and T2 values correlate inversely with each other in TLE cases but not in extratemporal/unclassified group. Visual assessment, volumetry, and T2 relaxometry showed equal efficacy in diagnosing unilateral pathology, but quantitative techniques are more sensitive in diagnosing bilateral pathologies and mild unilateral pathology. Among all modalities tested, hippocampal volumetry provided 75% lateralization and was most sensitive. Hippocampal volume ratio (HVR) and hippocampal volume difference (R-L) can be used to differentiate between temporal and extratemporal epilepsies except in cases with bilateral pathologies. No significant correlation exists between duration of seizures and hippocampal atrophy in both temporal and extratemporal groups of cases. 

## Figures and Tables

**Table:** (a)

Hippocampus	Number of patients
Hippocampal sclerosis	15
Hippocampal atrophy	1
Questionable hippocampal abnormality	1
Normal hippocampus	13

**Table:** (b)

	Left	Right	Bilateral
Increased signal on T2W images and atrophy	5	10	—
Atrophy without increased signal	1	—	—
Increased signal on T2W images without atrophy (questionable hippocampal abnormality)	1	—	—

**Table 2:** Quantitative hippocampal MRI data in controls and different groups.

Hippocampus	Control (*n* = 50)	Right TLE (*n* = 10)	Left TLE (*n* = 10)	Extratemporal/unclassified (*n* = 10)
(A) Volume				
Right				
Mean	3.73	2.23	3.69	3.53
SD	0.40	0.53	0.42	0.45
Range	2.97 to 4.28	1.40 to 2.80	3.10 to 4.40	2.60 to 4.00
Left				
Mean	3.60	3.31	2.79	3.39
SD	0.41	0.53	0.67	0.53
Range	2.90 to 4.50	2.70 to 4.20	2.10 to 4.00	2.50 to 4.20
(B) T2 Relaxometry				
Right				
Mean	112.58	173.08	122.81	113.41
SD	6.25	51.65	26.77	4.77
Range	101.00 to 125.60	111.30 to 269.50	107.00 to 196.70	106.40 to 123.20
Left				
Mean	112.12	116.90	135.76	115.88
SD	6.83	12.59	26.19	7.16
Range	102.00 to 125.60	104.30 to 147.90	110.40 to 201.80	103.50 to 125.70

**Table:** (a)

EEG localization	Number of patients having MRI findings in concordance with EEG		
	Visual analysis	Hippocampal volumetry	T2 relaxometry
Right TLE (*n* = 8)	8	8	8
Left TLE** (*n* = 8)	5	5	5
Bitemporal* (*n* = 4)	—	2	1

*EEG localized 4 cases as of bitemporal origin. Out of these 4 cases, 2 had dominant focus on right side and were grouped earlier under right TLE and 2 had dominant focus on left side and were grouped earlier under left TLE.

**Of the 8 left TLE patients (as localized by EEG), only 5 had MRI findings in concordance with EEG; the rest of the 3 patients had diagnosis of right MTS, FCD left parietal lobe, and calcified granuloma left centrum semiovale on MRI.

**Table:** (b)

	Visual analysis	Hippocampal volumetry	T2 relaxometry
Number of patients having MRI findings in concordance with EEG	13	15	14
Percentage concordance with EEG	65%	75%	70%

**Table:** (a)

Parameters	Controls (*n* = 50)	TLE (*n* = 20)	ET/UC (*n* = 10)
Mean	0.96	0.70	0.94
Range	0.91–1	0.47–0.97	0.76–1
SD	0.027	0.15	0.065

**Table:** (b)

R-L difference (cc)	Mean	0.155
	Range	0–0.3
	SD	0.105
